# Supplementary material for: Self-Reported Health Problems and Quality of Life in a Sample of Colombian Childhood Cancer Survivors: A Descriptive Cross-Sectional Study
Source: Cancers (Basel). 2022 Jun 18;14(12):2999. doi: 10.3390/cancers14122999 (PMC9221244; doi:10.3390/cancers14122999)
Supplement: Supplementary file 1 [file cancers-14-02999-s001.zip › Cancers-1651193_Informed consent_blank copy.pdf]

Consentimiento Informado

A continuación, solicitamos que diligencie el formato de consentimiento informado para poder participar en el estudio.

Introducción

Usted está siendo invitado a participar en el proyecto de investigación "Caracterización del estado de salud de una muestra de supervivientes colombianos de cáncer infantil". Este documento le proporciona la información necesaria para que usted participe voluntaria y libremente. Antes de dar su consentimiento, usted necesita entender plenamente el propósito de su decisión. Este proceso se denomina consentimiento informado. Una vez que haya leído este documento y resuelto con el investigador las dudas, se le pedirá que firme este formato de manera electrónica en señal de aceptación de participar.

Información general

1. ¿Por qué de debe realizar este estudio?

En Colombia, hasta hace poco, la supervivencia de cáncer infantil era muy baja, y el enfoque se había centrado principalmente en la atención curativa de este tipo de cáncer. Recientemente, las probabilidades de supervivencia han ido mejorando, particularmente para las leucemias y los linfomas, aunque todavía son menores comparadas con las de los países de altos ingresos. Sin embargo, las estrategias de seguimiento para caracterizar el estado de salud a largo plazo en estos supervivientes aún no se han abordado en nuestro país.

Debido a la temprana edad de aparición del cáncer infantil y la posible longevidad de estos niños y adolescentes, las consecuencias de posibles eventos adversos y/o comorbilidades pueden tener un mayor impacto en sus vidas y, en última instancia, en la sociedad en la cual van a vivir y van a ser parte.

Aunque en los países industrializados, la población de supervivientes de cáncer infantil se ha estudiado durante más de 30 años, en Colombia se desconoce el estado general de salud en esta población. Este estudio de corte transversal busca hacer una primera caracterización de esta población identificando por los diferentes sistemas (cardiovascular, pulmonar, neurológico, etc) la presencia de alteraciones en el estado de salud, comorbilidades y posibles secuelas a corto, mediano o largo plazo en una muestra de supervivientes colombianos de cáncer infantil.

2. ¿Cuál es el objetivo de este estudio?

Determinar la frecuencia de problemas de salud en una muestra de adultos colombianos supervivientes de cáncer infantil.

3. ¿En qué consiste el estudio?

Este estudio de corte transversal buscar investigar la presencia de potenciales problemas de salud entre adultos que tuvieron cáncer en su infancia. Para eso, se va a seleccionar una muestra de supervivientes de cáncer infantil utilizando varias estrategias (a través de la creación de una página web, a través de redes sociales, por contactos en hospitales con oncología pediátrica, entre otros...). Una vez se tengan seleccionados los participantes, se les enviará un cuestionario por correo electrónico en el cual se obtendrán datos generales acerca del diagnóstico de su enfermedad (cáncer infantil), el año de diagnóstico, generalidades del tratamiento recibido y se indagará sobre aspectos generales de su estado de salud de acuerdo a los diferentes sistemas (cardiovascular, pulmonar, neurológico, etc..). El cuestionario será contestado utilizando una plataforma digital (REDCap). Se dará la opción de contestar el cuestionario por vía telefónica, en caso de ser necesario. Se dará plazo de un mes para esperar que el participante conteste el cuestionario. Si al cabo de este mes no ha habido respuesta, se volverá a enviar el cuestionario.

4. ¿Cuáles son las molestias o los riesgos esperados?

Durante el desarrollo del cuestionario pueden abordarse temas sensibles para los participantes como lo es recordar la experiencia de haber tenido cáncer, los primeros síntomas, la angustia percibida de los padres, los dolores y molestias del tratamiento, el miedo de volver a enfermar, entre otros... Por eso se prevee contar con un(a) psicólogo(a) que podrá brindar apoyo emocional a los participantes en caso de ser necesario.

5. ¿Cuáles son los beneficios que puedo obtener por participar?

En general, el conocimiento adquirido de este estudio nos permitirá tener un primer acercamiento a esta población. Además, puede contribuir a mejorar la calidad de vida de estos niños, futuros adultos de diferentes maneras:

a. Permite a los médicos involucrados en el cuidado de supervivientes de cáncer infantil estar al tanto de problemas de salud específicos que pueden desarrollar a largo plazo, dar consejos, consultar a otros especialistas y, si es posible, comenzar un tratamiento apropiado y oportuno mediante la detección temprana de cualquier comorbilidad.

b. En el futuro, el conocimiento obtenido de este estudio podría contribuir al desarrollo e implementación de estrategias de seguimiento para los supervivientes de cáncer infantil, como formulación de política pública, con el apoyo de otras especialidades médicas como medicina familiar o medicina interna.

6. ¿Existe confidencialidad en el manejo de mis datos?

Este proyecto se acoge a la ley 1581 de 2012 (Hábeas Data) que aplica para el tratamiento de datos personales. La información recolectada será estrictamente confidencial. Su nombre no será utilizado en ningún informe cuando los resultados sean publicados. Sólo la investigadora principal del estudio tendrá acceso a la información recolectada.

7. ¿Existe alguna obligación financiera?

Participar en este estudio no tiene ningún costo económico para usted

8. ¿Cuánto tiempo durará mi participación en el estudio?

Su participación en el estudio corresponderá al tiempo que se tome en desarrollar el cuestionario, es decir entre 30 a 40 minutos. Posteriormente, al finalizar el estudio, le serán socializados los resultados.

9. ¿Qué sucede si no deseo participar o me retiro del estudio?

Su participación en el estudio es y será siempre voluntaria. Usted puede decidir no participar o retirarse en cualquier momento del estudio. No habrá ningún tipo de consecuencia con la decisión de dejar de participar.

10. Datos de contacto de investigadores y del Comité de Ética de la Investigación

En caso de que necesite información póngase en contacto con la investigadora principal del estudio Dra. Natalia Godoy Casasbuenas, teléfono:318-5581201, correo electrónico: natalia.godoy@javeriana.edu.co

Si tiene alguna duda sobre las consideraciones éticas de esta investigación que rigen a todos los centros participantes, podrá comunicarse con el Comité de Ética, Pontificia Universidad Javeriana, Dr. Carlos Gómez-Restrepo (Presidente) al teléfono 3208320 ext.2770

Autorización

He comprendido las explicaciones que en un lenguaje claro y sencillo se me han brindado. El investigador me ha permitido expresar todas mis observaciones y ha aclarado todas las dudas y preguntas que he planteado respecto a los fines, métodos, ventajas, inconvenientes y pronóstico de participar en el estudio. Se me ha proporcionado una copia de este documento.

Al firmar este documento doy mi consentimiento voluntario para participar en el estudio “Caracterización del estado de salud de una muestra de supervivientes colombianos de cáncer infantil”.

Participante

1) Nombre del participante ________________________________

2) Firma del participante ____

3) Tipo documento de identidad
   - ◯ RC
   - ◯ TI
   - ◯ CC
   - ◯ CE
   - ◯ PA

4) Número de documento de identidad ________________________________

5) Teléfono ________________________________

6) Fecha ________________________________

7) Certifico que toda la información en el documento anterior es correcta, y entiendo que firmar este formulario electrónicamente es el equivalente a firmar un documento físico.
   - ◯ No
   - ◯ Si

Miembro del equipo de investigación

8) Nombre del miembro del equipo de investigación que realiza el proceso de consentimiento ________________________________

9) Firma ____

10) Tipo documento de identidad
    - ◯ RC
    - ◯ TI
    - ◯ CC
    - ◯ CE
    - ◯ PA

11) Número de documento de identidad ________________________________

12) Fecha ________________________________

13) Rol en el proyecto ________________________________

14) Certifico que toda la información en el documento anterior es correcta, y entiendo que firmar este formulario electrónicamente es el equivalente a firmar un documento físico.

- ◯ No
- ◯ Si
