# Supplementary material for: Self-Reported Health Problems and Quality of Life in a Sample of Colombian Childhood Cancer Survivors: A Descriptive Cross-Sectional Study
Source: Cancers (Basel). 2022 Jun 18;14(12):2999. doi: 10.3390/cancers14122999 (PMC9221244; doi:10.3390/cancers14122999)
Supplement: Supplementary file 1 [file cancers-14-02999-s001.zip › Cancers-1651193_Study Questionnaire.pdf]

Consentimiento Informado

A continuación, solicitamos que diligencie el formato de consentimiento informado para poder participar en el estudio.

Introducción

Usted está siendo invitado a participar en el proyecto de investigación "Caracterización del estado de salud de una muestra de supervivientes colombianos de cáncer infantil". Este documento le proporciona la información necesaria para que usted participe voluntaria y libremente. Antes de dar su consentimiento, usted necesita entender plenamente el propósito de su decisión. Este proceso se denomina consentimiento informado. Una vez que haya leído este documento y resuelto con el investigador las dudas, se le pedirá que firme este formato de manera electrónica en señal de aceptación de participar.

Información general

1. ¿Por qué de debe realizar este estudio?

En Colombia, hasta hace poco, la supervivencia de cáncer infantil era muy baja, y el enfoque se había centrado principalmente en la atención curativa de este tipo de cáncer. Recientemente, las probabilidades de supervivencia han ido mejorando, particularmente para las leucemias y los linfomas, aunque todavía son menores comparadas con las de los países de altos ingresos. Sin embargo, las estrategias de seguimiento para caracterizar el estado de salud a largo plazo en estos supervivientes aún no se han abordado en nuestro país.

Debido a la temprana edad de aparición del cáncer infantil y la posible longevidad de estos niños y adolescentes, las consecuencias de posibles eventos adversos y/o comorbilidades pueden tener un mayor impacto en sus vidas y, en última instancia, en la sociedad en la cual van a vivir y van a ser parte.

Aunque en los países industrializados, la población de supervivientes de cáncer infantil se ha estudiado durante más de 30 años, en Colombia se desconoce el estado general de salud en esta población. Este estudio de corte transversal busca hacer una primera caracterización de esta población identificando por los diferentes sistemas (cardiovascular, pulmonar, neurológico, etc) la presencia de alteraciones en el estado de salud, comorbilidades y posibles secuelas a corto, mediano o largo plazo en una muestra de supervivientes colombianos de cáncer infantil.

2. ¿Cuál es el objetivo de este estudio?

Determinar la frecuencia de problemas de salud en una muestra de adultos colombianos supervivientes de cáncer infantil.

3. ¿En qué consiste el estudio?

Este estudio de corte transversal buscar investigar la presencia de potenciales problemas de salud entre adultos que tuvieron cáncer en su infancia. Para eso, se va a seleccionar una muestra de supervivientes de cáncer infantil utilizando varias estrategias (a través de la creación de una página web, a través de redes sociales, por contactos en hospitales con oncología pediátrica, entre otros...). Una vez se tengan seleccionados los participantes, se les enviará un cuestionario por correo electrónico en el cual se obtendrán datos generales acerca del diagnóstico de su enfermedad (cáncer infantil), el año de diagnóstico, generalidades del tratamiento recibido y se indagará sobre aspectos generales de su estado de salud de acuerdo a los diferentes sistemas (cardiovascular, pulmonar, neurológico, etc..). El cuestionario será contestado utilizando una plataforma digital (REDCap). Se dará la opción de contestar el cuestionario por vía telefónica, en caso de ser necesario. Se dará plazo de un mes para esperar que el participante conteste el cuestionario. Si al cabo de este mes no ha habido respuesta, se volverá a enviar el cuestionario.

4. ¿Cuáles son las molestias o los riesgos esperados?

Durante el desarrollo del cuestionario pueden abordarse temas sensibles para los participantes como lo es recordar la experiencia de haber tenido cáncer, los primeros síntomas, la angustia percibida de los padres, los dolores y molestias del tratamiento, el miedo de volver a enfermar, entre otros... Por eso se prevee contar con un(a) psicólogo(a) que podrá brindar apoyo emocional a los participantes en caso de ser necesario.

5. ¿Cuáles son los beneficios que puedo obtener por participar?

En general, el conocimiento adquirido de este estudio nos permitirá tener un primer acercamiento a esta población. Además, puede contribuir a mejorar la calidad de vida de estos niños, futuros adultos de diferentes maneras:

a. Permite a los médicos involucrados en el cuidado de supervivientes de cáncer infantil estar al tanto de problemas de salud específicos que pueden desarrollar a largo plazo, dar consejos, consultar a otros especialistas y, si es posible, comenzar un tratamiento apropiado y oportuno mediante la detección temprana de cualquier comorbilidad.

b. En el futuro, el conocimiento obtenido de este estudio podría contribuir al desarrollo e implementación de estrategias de seguimiento para los supervivientes de cáncer infantil, como formulación de política pública, con el apoyo de otras especialidades médicas como medicina familiar o medicina interna.

6. ¿Existe confidencialidad en el manejo de mis datos?

Este proyecto se acoge a la ley 1581 de 2012 (Hábeas Data) que aplica para el tratamiento de datos personales. La información recolectada será estrictamente confidencial. Su nombre no será utilizado en ningún informe cuando los resultados sean publicados. Sólo la investigadora principal del estudio tendrá acceso a la información recolectada.

7. ¿Existe alguna obligación financiera?

Participar en este estudio no tiene ningún costo económico para usted

8. ¿Cuánto tiempo durará mi participación en el estudio?

Su participación en el estudio corresponderá al tiempo que se tome en desarrollar el cuestionario, es decir entre 30 a 40 minutos. Posteriormente, al finalizar el estudio, le serán socializados los resultados.

9. ¿Qué sucede si no deseo participar o me retiro del estudio?

Su participación en el estudio es y será siempre voluntaria. Usted puede decidir no participar o retirarse en cualquier momento del estudio. No habrá ningún tipo de consecuencia con la decisión de dejar de participar.

10. Datos de contacto de investigadores y del Comité de Ética de la Investigación

En caso de que necesite información póngase en contacto con la investigadora principal del estudio Dra. Natalia Godoy Casasbuenas, teléfono:318-5581201, correo electrónico: natalia.godoy@javeriana.edu.co

Si tiene alguna duda sobre las consideraciones éticas de esta investigación que rigen a todos los centros participantes, podrá comunicarse con el Comité de Ética, Pontificia Universidad Javeriana, Dr. Carlos Gómez-Restrepo (Presidente) al teléfono 3208320 ext.2770

Autorización

He comprendido las explicaciones que en un lenguaje claro y sencillo se me han brindado. El investigador me ha permitido expresar todas mis observaciones y ha aclarado todas las dudas y preguntas que he planteado respecto a los fines, métodos, ventajas, inconvenientes y pronóstico de participar en el estudio. Se me ha proporcionado una copia de este documento.

Al firmar este documento doy mi consentimiento voluntario para participar en el estudio “Caracterización del estado de salud de una muestra de supervivientes colombianos de cáncer infantil”.

Participante

1) Nombre del participante ________________________________

2) Firma del participante ________________________________

3) Tipo documento de identidad
- ◯ RC
- ◯ TI
- ◯ CC
- ◯ CE
- ◯ PA

4) Número de documento de identidad ________________________________

5) Teléfono ________________________________

6) Fecha ________________________________

7) Certifico que toda la información en el documento anterior es correcta, y entiendo que firmar este formulario electrónicamente es el equivalente a firmar un documento físico.
- ◯ No
- ◯ Si

Miembro del equipo de investigación

8) Nombre del miembro del equipo de investigación que realiza el proceso de consentimiento ________________________________

9) Firma ________________________________

10) Tipo documento de identidad
- ◯ RC
- ◯ TI
- ◯ CC
- ◯ CE
- ◯ PA

11) Número de documento de identidad ________________________________

12) Fecha ________________________________

13) Rol en el proyecto ________________________________

14) Certifico que toda la información en el documento anterior es correcta, y entiendo que firmar este formulario electrónicamente es el equivalente a firmar un documento físico.

◯ No
◯ Si

Estudio Lázaro

Caracterización del estado de salud en una muestra de supervivientes de cáncer infantil colombianos

---

Muchas gracias por participar en el proyecto LÁZARO. Para este estudio te pediremos llenar un cuestionario.

En la mayoría de preguntas te pediremos seleccionar tu respuesta de una lista de opciones. Sin embargo, hay otras en las que te pediremos que escribas tus respuestas.

Recuerda que toda la información que completes en este cuestionario es confidencial, solo será usada para esta investigación y no será compartida con personas fuera del equipo de investigación.

Para comenzar diligencia tus datos de identificación y luego presiona "Submit".

---

1) Nombre ________________________________

2) Apellido ________________________________

3) Fecha de evaluación ________________________________

Preguntas acerca de ti

Q1. Por favor marca tus respuestas o escríbelas en los espacios correspondientes:

1.1 Fecha de nacimiento	__________________________
1.2 ¿Cómo describirías tu género?	◯ Masculino<br>◯ Femenino<br>◯ Otro
1.3 ¿Cuál es tu estado civil?	◯ Soltero(a)<br>◯ Casado(a)<br>◯ Unión libre<br>◯ Separado(a)<br>◯ Divorciado(a)<br>◯ Viudo(a)
1.4 ¿Tienes hijos(as)?	◯ Sí<br>◯ No
1.5 ¿Cuántos hijos(as) tienes?	__________________________
1.6 ¿Cuál es el nivel de educación más alto que has completado?<br>(Elija solo una opción de respuesta)	◯ No tengo educación formal<br>◯ He completado educación primaria o menos<br>◯ He completado educación secundaria<br>◯ He completado educación técnica<br>◯ He completado educación universitaria (pregrado)<br>◯ He completado educación universitaria (postgrado)<br>◯ Otros
Por favor especifica cuál:	__________________________
1.7 ¿Cuál de las siguientes opciones describe mejor tu ocupación principal actual?<br>(Elija solo una opción de respuesta)	◯ Trabajo<br>◯ Estudio (en el colegio, educación técnica/universitaria)<br>◯ Ama(o) de casa<br>◯ Otros (especificar)
Por favor especifica cuál:	__________________________
1.8 ¿ Actualmente estás afiliado, eres cotizante o eres beneficiario de alguna entidad de seguridad social en salud?<br>(Entidad Promotora de Salud -EPS, Administradora de Régimen Subsidiado -ARS, o a través del SISBEN)	◯ Si<br>◯ No<br>◯ No sé
1.9 En caso de ser positivo, ¿a cuál de los siguientes regímenes de Seguridad Social en Salud estás afiliado?	◯ Contributivo/EPS<br>◯ Especial (Fuerzas Armadas, Ecopetrol, Universidades públicas, Magisterio)<br>◯ Subsidiado/ SISBEN<br>◯ No sé
1.9b ¿Tuviste alguna dificultad para acceder a este régimen de Seguridad Social en Salud?	◯ Si<br>◯ No

1.10 ¿Actualmente estás afiliado a un seguro de vida?	◯ Si<br>◯ No
1.10b En caso de ser positivo, ¿tuviste alguna dificultad para acceder a este seguro de vida?	◯ Si<br>◯ No
1.11 ¿Cuántos hermano(as) biológico(as) (mismo padre y madre) tienes?	◯ Uno<br>◯ Dos<br>◯ Tres<br>◯ Más de tres<br>◯ No tengo hermanos
1.12 Peso actual (en kg)	______________________
1.13 Estatura actual (en cm)	______________________

5% Completado

Presiona "Submit" para continuar.

Cuidado médico

Las siguientes preguntas son acerca del cuidado médico que recibiste a partir del diagnóstico del cáncer que presentaste durante tu infancia o adolescencia

2.1 ¿Cuáles de las siguientes enfermedades te fue diagnosticada durante tu infancia/adolescencia?	◯ Leucemia<br>◯ Linfoma<br>◯ Tumor cerebral<br>◯ Osteosarcoma<br>◯ Otro (especificar)
Por favor especifica cuál:	______________________________
2.2 ¿Sabes en qué estadío fue identificada tu enfermedad?	◯ Temprano<br>◯ Avanzado<br>◯ No sé/ No recuerdo
2.3 ¿Cuántos años tenías al momento del diagnóstico de tu enfermedad?	______________________________
2.4 ¿En qué institución recibiste tratamiento para tu enfermedad?<br>(En caso de no saber, por favor indica "no sé")	______________________________
2.5 ¿Durante cuántos años estuviste en tratamiento?	◯ Un año<br>◯ Dos años<br>◯ Tres años<br>◯ Más de tres años
2.6 ¿Qué tipo de tratamiento recibiste para tu enfermedad?<br>(Puedes escoger más de una respuesta)	☐ Quimioterapia<br>☐ Radioterapia<br>☐ Cirugia<br>☐ Otro
2.6b En caso de haber recibido otro tipo de tratamiento, por favor especifica cual:	______________________________
2.7 ¿Alguna vez estuviste hospitalizado por presentar complicaciones debidas al tratamiento?	◯ Si<br>◯ No
2.8 En caso de ser positivo, especifica ¿cuántas veces?	◯ Una vez<br>◯ Dos veces<br>◯ Tres veces<br>◯ Más de tres veces<br>◯ Otro
2.9 ¿Alguna vez estuviste hospitalizado en la Unidad de Cuidado Intensivo?	◯ Si<br>◯ No<br>◯ No sé/ no recuerdo
2.10 En caso de ser positivo, especifica ¿cuántos días estuviste hospitalizado/a?	◯ Menos de tres días<br>◯ De tres a siete días<br>◯ Más de 8 días

2.11 En el momento actual, presentas alguna de las siguientes condiciones?

	Si	No
¿Pérdida persistente del cabello?	◯	◯
¿Cicatrices o malformación de la cabeza o del cuello (incluyendo la cara)?	◯	◯
¿Cicatrices o malformación del pecho o del abdomen?	◯	◯
¿Cicatrices o malformación de los brazos o piernas (incluyendo un brazo o una pierna anormalmente más corta?	◯	◯
¿Caminas cojeando?	◯	◯
¿Pérdida de un brazo, una pierna, un dedo de la mano o del pie?	◯	◯
¿Pérdida de un ojo?	◯	◯

10% Completado

Presiona "Submit" para continuar.

Condiciones médicas posterior al tratamiento

La siguiente serie de preguntas están relacionadas con condiciones médicas que te hayan ocurrido alguna vez en tu vida, posterior al tratamiento recibido para el cáncer que te fue diagnosticado durante la infancia o adolescencia.

Por favor indica, seleccionando con un círculo ("No", "Sí" o "No sé/No estoy seguro") si un médico u otro profesional de la salud te ha dicho que tienes alguna de las siguientes condiciones.

Además, indica la edad aproximada que tenías cuando se te informó por primera vez sobre esta condición.

Para empezar presiona "Submit"

A-Sistema auditivo/visión/habla

¿Alguna vez tu médico u otro profesional de la salud te ha dicho que presentas alguna de las siguientes condiciones médicas?

A1. ¿Pérdida de la audición requiriendo de alguna ayuda audiológica?	◯ Si<br>◯ No<br>◯ No sé /no estoy seguro(a)
En caso de ser positivo, indica la edad aproximada cuando se te informó por primera vez sobre esta condición:	______________________
A2. ¿Sordera en un oído o ambos?	◯ Si<br>◯ No<br>◯ No sé /no estoy seguro(a)
En caso de ser positivo, indica la edad aproximada cuando se te informó por primera vez sobre esta condición:	______________________
A3. ¿Presencia de un sonido constante (tinnitus) en los oídos?	◯ Si<br>◯ No<br>◯ No sé /no estoy seguro(a)
En caso de ser positivo, indica la edad aproximada cuando se te informó por primera vez sobre esta condición:	______________________
A4. ¿Sensación de mareo o vértigo persistente?	◯ Si<br>◯ No<br>◯ No sé /no estoy seguro(a)
En caso de ser positivo, indica la edad aproximada cuando se te informó por primera vez sobre esta condición:	______________________
A5. ¿Cualquier otra condición relacionada con la audición?	◯ Si<br>◯ No
En caso de ser positivo, especifica cuál:	______________________
Por favor especifica la edad aproximada cuando se te informó por primera vez sobre esta condición:	______________________
A6. ¿Ceguera en uno o ambos ojos?	◯ Si<br>◯ No<br>◯ No sé /no estoy seguro(a)
En caso de ser positivo, indica la edad aproximada cuando se te informó por primera vez sobre esta condición:	______________________
A7. ¿Presencia de cataratas en uno o ambos ojos?	◯ Si<br>◯ No<br>◯ No sé /no estoy seguro(a)

En caso de ser positivo, indica la edad aproximada cuando se te informó por primera vez sobre esta condición:	__________________________
A8. ¿Algún problema de visión en uno o ambos ojos aún utilizando gafas?	◯ Si<br>◯ No<br>◯ No sé /no estoy seguro(a)
En caso de ser positivo, indica la edad aproximada cuando se te informó por primera vez sobre esta condición:	__________________________
A9. ¿Presencia de ojo seco requiriendo de gotas lubricantes?	◯ Si<br>◯ No<br>◯ No sé /no estoy seguro(a)
En caso de ser positivo, indica la edad aproximada cuando se te informó por primera vez sobre esta condición:	__________________________
A10. ¿Cualquier otra condición relacionada con la visión?	◯ Si<br>◯ No<br>◯ No sé /no estoy seguro(a)
En caso de ser positivo, especifica cual:	__________________________
Por favor especifica la edad aproximada cuando se te informó por primera vez sobre esta condición:	__________________________
A11. ¿Algún defecto de masticación?	◯ Si<br>◯ No<br>◯ No sé /no estoy seguro(a)
En caso de ser positivo, indica la edad aproximada cuando se te informó por primera vez sobre esta condición:	__________________________
A12. ¿Algún defecto de fonación (emisión de la voz o de la palabra)?	◯ Si<br>◯ No<br>◯ No sé /no estoy seguro(a)
En caso de ser positivo, indica la edad aproximada cuando se te informó por primera vez sobre esta condición:	__________________________
A13. ¿Algún defecto en la deglución (paso de alimentos o bebidas de la boca al estómago)?	◯ Si<br>◯ No<br>◯ No sé /no estoy seguro(a)
En caso de ser positivo, indica la edad aproximada cuando se te informó por primera vez sobre esta condición:	__________________________
A14. ¿Alguna vez has presentado pérdida del olfato que haya durado tres meses o más?	◯ Si<br>◯ No<br>◯ No sé /no estoy seguro(a)

En caso de ser positivo, indica la edad aproximada cuando se te informó por primera vez sobre esta condición: ______________________

15% Completado

Presiona "Submit" para continuar.

B- Sistema dental

¿Alguna vez tu médico u otro profesional de la salud te ha dicho que presentas alguna de las siguientes condiciones médicas?

B.1 ¿Has sufrido malformaciones en los dientes (hipodoncia, microdoncia) o mandíbula (maloclusión) que haya necesitado tratamiento odontológico?	◯ Si<br>◯ No<br>◯ No estoy seguro(a)
En caso de ser positivo, indica la edad aproximada cuando se te informó por primera vez sobre esta condición:	__________________
B.2 ¿Has presentado disminución en la producción de saliva?	◯ Si<br>◯ No<br>◯ No estoy seguro(a)
En caso de ser positivo, indica la edad aproximada cuando se te informó por primera vez sobre esta condición:	__________________

20% Completado

Presiona "Submit" para continuar.

C- Piel y anexos

¿Alguna vez tu médico u otro profesional de la salud te ha dicho que presentas alguna de las siguientes condiciones médicas?

C.1 ¿Has presentado problemas o manchas en las uñas?	◯ Si<br>◯ No<br>◯ No estoy seguro(a)
En caso de ser positivo, indica la edad aproximada cuando se te informó por primera vez sobre esta condición:	______________________
C.2 ¿Has presentado caída abundante de cabello?	◯ Si<br>◯ No<br>◯ No estoy seguro(a)
En caso de ser positivo, indica la edad aproximada cuando se te informó por primera vez sobre esta condición:	______________________
C.3 ¿Has presentado carencia de cabello?	◯ Si<br>◯ No<br>◯ No estoy seguro(a)
En caso de ser positivo, indica la edad aproximada cuando se te informó por primera vez sobre esta condición:	______________________

25% Completado

Presiona "Submit" para continuar.

D - Sistema Urinario

¿Alguna vez tu médico u otro profesional de la salud te ha dicho que presentas alguna de las siguientes condiciones médicas?

D.1 ¿Infecciones en el riñón a repetición?	◯ Si<br>◯ No<br>◯ No estoy seguro(a)
En caso de ser positivo, indica la edad aproximada cuando se te informó por primera vez sobre esta condición:	________________________
D.2 ¿Cálculos (piedras) en los riñones?	◯ Si<br>◯ No<br>◯ No estoy seguro(a)
En caso de ser positivo, indica la edad aproximada cuando se te informó por primera vez sobre esta condición:	________________________
D.3 ¿Infecciones urinarias a repetición?	◯ Si<br>◯ No<br>◯ No estoy seguro(a)
En caso de ser positivo, indica la edad aproximada cuando se te informó por primera vez sobre esta condición:	________________________
D.4 ¿Actualmente estás en diálisis?	◯ Si<br>◯ No<br>◯ No estoy seguro(a)
En caso de ser positivo, indica la edad aproximada cuando se te informó por primera vez sobre esta condición:	________________________
D.5 ¿Sufres de cualquier otra condición relacionada con los riñones o el tracto urinario?	◯ Si<br>◯ No
En caso de ser positivo, especifica cuál:	________________________
Por favor indica la edad aproximada que tenías cuando se te informó acerca de esa condición:	________________________

30% Completado

Presiona "Submit" para continuar.

E - Sistema Endocrinológico

¿Alguna vez tu médico u otro profesional de la salud te ha dicho que presentas alguna de las siguientes condiciones médicas?

E.1 ¿Problemas de obesidad o sobrepeso?	◯ Si<br>◯ No<br>◯ No estoy seguro(a)
En caso de ser positivo, indica la edad aproximada cuando se te informó por primera vez sobre esta condición:	__________________________
E.2 ¿Diabetes (azúcar elevado en sangre)?	◯ Si<br>◯ No<br>◯ No estoy seguro(a)
En caso de ser positivo, indica la edad aproximada cuando se te informó por primera vez sobre esta condición:	__________________________
E.2b ¿Qué tratamiento utiliza para el manejo de la diabetes?	◯ Solo dieta<br>◯ Medicamentos orales<br>◯ Medicamentos inyectados
E.3 ¿Dislipidemia (grasas elevadas en sangre)?	◯ Si<br>◯ No<br>◯ No sé
En caso de ser positivo, indica la edad aproximada cuando se te informó por primera vez sobre esta condición:	__________________________
E.4 ¿Tomas algún medicamento para la tiroides?	◯ Si<br>◯ No<br>◯ No estoy seguro(a)
En caso de ser positivo, especifica cuál:	__________________________
E.5 ¿Has recibido alguna vez hormona del crecimiento?	◯ Si<br>◯ No<br>◯ No estoy seguro(a)
En caso de ser positivo, especifica la edad en que la recibiste:	__________________________
E.6 ¿Alguna vez te han aplicado algún medicamento para el tratamiento contra la osteoporosis?	◯ Si<br>◯ No<br>◯ No estoy seguro(a)
En caso de ser positivo, especifica la edad en que lo recibiste:	__________________________

35% Completado

Presiona "Submit" para continuar.

F - Sistema reproductor

¿Alguna vez tu médico u otro profesional de la salud te ha dicho que presenta alguna de las siguientes condiciones médicas?

F.1 ¿Alguna vez tu médico te ha dicho que podrías tener dificultades para tener hijos?	◯ Si<br>◯ No<br>◯ No estoy seguro(a)
En caso de ser positivo, indica la edad aproximada cuando se te informó por primera vez sobre esta condición:	________________
F.2 ¿Alguna vez te has hecho exámenes médicos (como un análisis de sangre, una ecografía o conteo de espermatozoides) para ver si podrías tener problemas para tener hijos?	◯ Si<br>◯ No<br>◯ No estoy seguro(a)
En caso de ser positivo, indica la edad aproximada cuando te realizaste alguno de esos exámenes:	________________
F.3 ¿Alguna vez te han dicho si tienes un bajo conteo de espermatozoides?	◯ Si<br>◯ No<br>◯ No estoy seguro
En caso de ser positivo, indica la edad aproximada cuando se te informó por primera vez sobre esta condición:	________________
F.4 ¿Alguna vez has tenido un periodo menstrual en tu vida?	◯ Si<br>◯ No<br>◯ No estoy segura
En caso de ser positivo, indica la edad aproximada de tu primer periodo menstrual:	________________
F.5 ¿Tienes actualmente tus periodos menstruales?	◯ Si<br>◯ No<br>◯ No estoy segura
En caso de ser negativo, indica la edad aproximada de tu último periodo:	________________
F.6 ¿Cuál de los siguientes métodos anticonceptivos utilizas actualmente?	◯ Anticonceptivos orales<br>◯ Métodos de barrera (condón)<br>◯ Dispositivo intra uterino (T)<br>◯ Ligadura de trompas<br>◯ Vasectomía<br>◯ Ninguno<br>◯ Otro
Por favor, especifica cuál:	________________

40% Completado

Presiona "Submit" para continuar.

G - Sistema Cardiovascular

¿Alguna vez tu médico u otro profesional de la salud te ha dicho que presenta alguna de las siguientes condiciones médicas?

G.1 ¿Arritmias (palpitaciones) que hayan requerido de medicamentos o seguimiento?	◯ Si<br>◯ No<br>◯ No estoy seguro(a)
En caso de ser positivo, indica la edad aproximada cuando se te informó por primera vez sobre esta condición:	______________________
G.2 ¿Insuficiencia cardiaca (músculo cardiaco débil)?	◯ Si<br>◯ No<br>◯ No estoy seguro(a)
En caso de ser positivo, indica la edad aproximada cuando se te informó por primera vez sobre esta condición:	______________________
G.3 ¿Infarto agudo de miocardio (ataque cardiaco) o enfermedad coronaria?	◯ Si<br>◯ No<br>◯ No estoy seguro(a)
En caso de ser positivo, indica la edad aproximada cuando se te informó por primera vez sobre esta condición:	______________________
G.4 ¿Hipertensión (tensión alta) que haya requerido medicación?	◯ Si<br>◯ No<br>◯ No estoy seguro(a)
En caso de ser positivo, indica la edad aproximada cuando se te informó por primera vez sobre esta condición:	______________________
G.5 ¿Evento cerebro vascular (derrame cerebral)?	◯ Si<br>◯ No<br>◯ No estoy seguro(a)
En caso de ser positivo, indica la edad aproximada cuando se te informó por primera vez sobre esta condición:	______________________
G.6 ¿Dolor intenso en el pecho (angina)?	◯ Si<br>◯ No<br>◯ No estoy seguro(a)
En caso de ser positivo, indica la edad aproximada cuando se te informó por primera vez sobre esta condición:	______________________
G.7 ¿Hacer ejercicio te genera dolor en el pecho, falta de aire o ritmo cardiaco irregular?	◯ Si<br>◯ No<br>◯ No estoy seguro(a)

En caso de ser positivo, indica la edad aproximada cuando se te informó por primera vez sobre esta condición:	__________________________
G.8 ¿Alguna vez has ido a consulta con un cardiólogo (especialista del corazón)?	◯ Si<br>◯ No<br>◯ No estoy seguro(a)
En caso de ser positivo, indica la edad aproximada cuando asististe donde un cardiólogo:	__________________________
G.9 ¿Algún miembro de la familia (madre, padre, hermano(a)s) ha tenido alguna vez un infarto?	◯ Si<br>◯ No<br>◯ No estoy seguro(a)
G.10 ¿Cualquier otra condición relacionada con el corazón o la circulación?	◯ Si<br>◯ No
En caso de ser positivo, especifica cuál:	__________________________
Por favor indica la edad aproximada cuando se te informó por primera vez sobre esta condición:	__________________________

45% Completado

Presiona "Submit" para continuar.

H - Sistema Respiratorio

¿Alguna vez tu médico u otro profesional de la salud te ha dicho que presentas alguna de las siguientes condiciones médicas?

H.1 ¿Presencia de asma?	◯ Si<br>◯ No<br>◯ No estoy seguro(a)
En caso de ser positivo, indica la edad aproximada cuando se te informó por primera vez sobre esta condición:	__________________
H.2 ¿Actualmente utilizas oxígeno ambulatorio?	◯ Si<br>◯ No<br>◯ No estoy seguro(a)
En caso de ser positivo, indica la edad aproximada cuando empezaste a usar oxígeno:	__________________
H.3 ¿Presencia de neumonía recurrente (más de 2-3 veces en el último año)?	◯ Si<br>◯ No<br>◯ No estoy seguro(a)
En caso de ser positivo, indica la edad aproximada cuando se te informó por primera vez sobre esta condición:	__________________
H.4 ¿Enfisema?	◯ Si<br>◯ No<br>◯ No estoy seguro(a)
En caso de ser positivo, indica la edad aproximada cuando se te informó por primera vez sobre esta condición:	__________________
H.5 ¿Fibrosis pulmonar?	◯ Si<br>◯ No<br>◯ No estoy seguro(a)
En caso de ser positivo, indica la edad aproximada cuando se te informó por primera vez sobre esta condición:	__________________
H.6 ¿Alguna vez has ido a consulta con un neumólogo (especialista de los pulmones)?	◯ Si<br>◯ No<br>◯ No estoy seguro(a)
En caso de ser positivo, indica la edad aproximada cuando asististe donde un neumólogo:	__________________
H.7 ¿Cualquier otra condición relacionada con la respiración o los pulmones?	◯ Si<br>◯ No
En caso de ser positivo, especifica cuál:	__________________

Por favor indica la edad aproximada que tenías cuando se te informó por primera vez sobre esta condición: __________________________

50% Completado

Presiona "Submit" para continuar.

I - Sistema Digestivo

¿Alguna vez tu médico u otro profesional de la salud te ha dicho que presentas alguna de las siguientes condiciones médicas?

I.1 ¿Cirrosis hepática?	◯ Si<br>◯ No<br>◯ No estoy seguro(a)
En caso de ser positivo, indica la edad aproximada cuando se te informó por primera vez sobre esta condición:	__________________
I.2 ¿Hepatitis?	◯ Si<br>◯ No<br>◯ No estoy seguro(a)
En caso de ser positivo, indica la edad aproximada cuando se te informó por primera vez sobre esta condición:	__________________
I.3 ¿Alguna enfermedad del esófago?	◯ Si<br>◯ No<br>◯ No estoy seguro(a)
En caso de ser positivo, indique la edad aproximada cuando se le informó por primera vez sobre esta condición:	__________________
I.4 ¿Gastritis (dolor en la boca del estómago, pirosis, agrieras)?	◯ Si<br>◯ No<br>◯ No sé
En caso de ser positivo, indica la edad aproximada cuando se te informó por primera vez sobre esta condición:	__________________
I.5 ¿Diarrea crónica?	◯ Si<br>◯ No<br>◯ No estoy seguro(a)
En caso de ser positivo, indica la edad aproximada cuando se te informó por primera vez sobre esta condición:	__________________
I.6 ¿Alguna vez has tenido una gastrostomía?	◯ Si<br>◯ No<br>◯ No estoy seguro(a)
En caso de ser positivo, indica la edad cuando te la pusieron:	__________________
En caso de ser positivo, todavía la tienes?	◯ Si<br>◯ No

I.7 ¿Alguna vez has ido a consulta con un gastroentérologo (especialista del estómago/intestinos)?	◯ Si<br>◯ No<br>◯ No estoy seguro(a)
En caso de ser positivo, indica la edad aproximada cuando asististe donde un gastroenterólogo:	__________________________
I.8 ¿Cualquier otra condición relacionada con el sistema digestivo?	◯ Si<br>◯ No
En caso de ser positivo, especifica cuál:	__________________________
Por favor indica la edad aproximada que tenías cuando se te informó por primera vez sobre esta condición:	__________________________

55% Completado

Presiona "Submit" para continuar.

J - Cerebro y Sistema Nervioso

¿Alguna vez tu médico u otro profesional de la salud te ha dicho que presentas alguna de las siguientes condiciones médicas?

J.1 ¿Dolores de cabeza frecuentes?	◯ Si<br>◯ No<br>◯ No estoy seguro(a)
En caso de ser positivo, indica la edad aproximada cuando se te informó por primera vez sobre esta condición:	__________________
J.2 ¿Convulsiones?	◯ Si<br>◯ No<br>◯ No estoy seguro(a)
En caso de ser positivo, indica la edad aproximada cuando se te informó por primera vez sobre esta condición:	__________________
J.3 ¿Problemas con el equilibrio?	◯ Si<br>◯ No<br>◯ No estoy seguro(a)
En caso de ser positivo, indica la edad aproximada cuando se te informó por primera vez sobre esta condición:	__________________
J.4 ¿Problemas de concentración y falta de memoria?	◯ Si<br>◯ No<br>◯ No estoy seguro(a)
En caso de ser positivo, indica la edad aproximada cuando se te informó por primera vez sobre esta condición:	__________________
J.5 ¿Alguna vez has ido a consulta con un neurólogo (especialista del sistema nervioso)?	◯ Si<br>◯ No<br>◯ No estoy seguro(a)
En caso de ser positivo, indica la edad aproximada cuando asististe donde un neurólogo:	__________________
J.6 ¿Cualquier otra condición relacionada con el cerebro o sistema nervioso?	◯ Si<br>◯ No
En caso de ser positivo, especifica cuál:	__________________
Por favor indica la edad aproximada que tenías cuando se te informó por primera vez sobre esta condición:	__________________

60% Completado

IV - Procedimientos Quirúrgicos

En las siguientes preguntas, por favor indique si alguna vez le han realizado alguno de los siguientes procedimientos quirúrgicos. En caso afirmativo, indique la edad aproximada cuando se realizó esta cirugía.

4.1 ¿Amputación de una pierna, brazo, mano, pie o dedo?	◯ Si<br>◯ No<br>◯ No estoy seguro(a)
En caso de ser positivo, indica la edad aproximada cuando te realizaron esa cirugía:	__________________________________
4.2 ¿Procedimientos de alargamiento o acortamiento de las piernas?	◯ Si<br>◯ No<br>◯ No estoy seguro(a)
En caso de ser positivo, indica la edad aproximada cuando te realizaron esa cirugía:	__________________________________
4.3 ¿Cirugía de reemplazo articular?	◯ Si<br>◯ No<br>◯ No estoy seguro(a)
En caso de ser positivo, indica la edad aproximada cuando te realizaron esa cirugía:	__________________________________
4.4 ¿Cualquier otra cirugía de los huesos?	◯ Si<br>◯ No
En caso de ser positivo, especifica cuál:	__________________________________
Por favor indica la edad aproximada que tenías cuando te realizaron esa cirugía:	__________________________________
4.5 ¿Cirugía de bypass coronario?	◯ Si<br>◯ No<br>◯ No estoy seguro(a)
En caso de ser positivo, indica la edad aproximada cuando te realizaron esa cirugía:	__________________________________
4.6 ¿Angioplastia (cateterismos cardiacos o implantes de stent en arterias del corazón)?	◯ Si<br>◯ No<br>◯ No estoy seguro(a)
En caso de ser positivo, indica la edad aproximada cuando te realizaron esa cirugía:	__________________________________
4.7 ¿Cualquier otra cirugía del corazón?	◯ Si<br>◯ No
En caso de ser positivo, especifica cuál:	__________________________________
Por favor indica la edad aproximada que tenías cuando te realizaron esa cirugía:	__________________________________

4.8 ¿Colostomía o ilieostomía?	◯ Si<br>◯ No<br>◯ No estoy seguro(a)
En caso de ser positivo, indica la edad aproximada cuando te realizaron esa cirugía:	__________________________
4.9 ¿Esplenectomía?	◯ Si<br>◯ No<br>◯ No estoy seguro(a)
En caso de ser positivo, indica la edad aproximada cuando te realizaron esa cirugía:	__________________________
4.10 ¿Cirugía de seno para la extracción o biopsia de una masa sospechosa?	◯ Si<br>◯ No<br>◯ No estoy seguro(a)
En caso de ser positivo, indica la edad aproximada cuando te realizaron esa cirugía:	__________________________
4.11 ¿Broncoscopia desde que terminó el tratamiento para el cáncer infantil o adolescencia?	◯ Si<br>◯ No<br>◯ No estoy seguro(a)
En caso de ser positivo, indica la edad aproximada cuando te realizaron esa cirugía:	__________________________
4.12 ¿Una biopsia hepática desde que terminaste el tratamiento para el cáncer infantil?	◯ Si<br>◯ No<br>◯ No estoy seguro(a)
En caso de ser positivo, indica la edad aproximada cuando te realizaron esa cirugía:	__________________________
4.13 ¿Ha recibido algún trasplante?	◯ Trasplante de médula ósea<br>◯ Trasplante de corazón<br>◯ Trasplante de pulmón<br>◯ Trasplante de riñón<br>◯ Otro<br>◯ No he recibido ningún trasplante
En caso de ser positivo, por favor indica la edad aproximada cuando te realizaron ese trasplante:	__________________________
Por favor especifica cuál y a qué edad:	__________________________
4.14 ¿Cualquier otra cirugía?	◯ Si<br>◯ No
En caso de ser positivo, especifica cuál:	__________________________
Por favor indica la edad aproximada que tenías cuando te realizaron esa cirugía:	__________________________

65% Completado

V - Segundas Neoplasias

Las siguientes preguntas están relacionadas con el diagnóstico de otro cáncer, leucemia, tumores u otra enfermedad similar, o una recaída de tu diagnóstico inicial, que haya ocurrido desde el primer cáncer.

5.1 ¿En algún momento posterior al diagnóstico original de tu cáncer, has sido diagnosticado con otro cáncer, leucemia, tumor o similares? (Incluye recaída del diagnóstico original)

◯ Si
◯ No

5.2 En caso de ser positivo, escribe el nombre de la enfermedad: __________________________________

5.3 ¿Dónde se hizo el diagnóstico de este segundo cáncer? Por favor indica:

5.3a Nombre del hospital: __________________________________

5.3b Ciudad: __________________________________

5.3c Departamento: __________________________________

5.3d Nombre del médico: __________________________________

5.4 Este segundo cáncer fue:

◯ Recaída del diagnóstico original
◯ Nuevo cáncer, leucemia, tumor o enfermedad similar
◯ No sé

5.5 Fecha de diagnóstico del segundo cáncer:
En caso de no recordar la fecha exacta por favor coloca el 1er día del mes más cercano a la fecha de diagnóstico

____

5.6 Has tenido algún cáncer, leucemia, tumor adicional después del segundo cáncer

◯ Si
◯ No

5.7 En caso de ser positivo, escribe el nombre de la enfermedad: __________________________________

5.8 ¿Dónde se hizo el diagnóstico de este tercer cáncer? Por favor indica:

5.8a Nombre del hospital: __________________________________

5.8b Ciudad: __________________________________

5.8c Departamento: __________________________________

5.8d Nombre del médico: __________________________________

5.9 Este tercer cáncer fue:	◯ Recaída del diagnóstico original<br>◯ Nuevo cáncer, leucemia, tumor o enfermedad similar<br>◯ No sé
5.10 Fecha del diagnóstico del tercer cáncer:<br>En caso de no recordar la fecha exacta por favor coloca el 1er día del mes más cercano a la fecha de diagnóstico	______________________________

70% Completado

VI - Hábitos Saludables

Por favor responde las siguientes preguntas sobre tus hábitos saludables.

6.1 ¿Practicas alguna actividad física regularmente?	◯ Si<br>◯ No
6.2 En caso de ser positivo, cuántas veces a la semana practicas ejercicio?	◯ Menos de una vez a la semana<br>◯ Una vez a la semana<br>◯ Dos veces a la semana<br>◯ Tres veces a la semana<br>◯ Cuatro o más veces a la semana
6.3 Si no practicas alguna actividad física regularmente, ¿cuáles son las razones para no hacerlo?	☐ No tengo suficiente tiempo<br>☐ Es muy costoso<br>☐ Tengo problemas de salud que me impiden participar<br>☐ No hay instalaciones para hacer deporte cerca de donde vivo<br>☐ No me interesan los deportes<br>☐ Otros
6.4 Alguna vez has fumado en tu vida?	◯ Si<br>◯ No
6.5 Actualmente fumas?	◯ Si<br>◯ No
6.6 En caso de ser positivo, cuántos cigarrillos fumas al día?	◯ 0-4<br>◯ 5-9<br>◯ 10-15<br>◯ Más de 15
6.7¿A qué edad empezaste a fumar?	__________________________
6.8 ¿Alguna vez has tomado alcohol?	◯ Si<br>◯ No
6.9 En caso de ser positivo, ¿cuántos tragos de alcohol te tomas a la semana?<br>(un trago equivale a una copa de vino, una cerveza, etc..)	◯ 0 - 4<br>◯ 5 - 9<br>◯ 10 - 15<br>◯ Más de 15
6.10 ¿A qué edad empezaste a tomar alcohol?	__________________________

75% Completado

VII - Estado general de salud

Por favor responda las siguientes preguntas sobre su estado general de salud.

7.1 ¿Dirías que tu estado actual de salud es?	◯ Excelente<br>◯ Muy bueno<br>◯ Bueno<br>◯ Aceptable<br>◯ Malo
7.2 Algunas personas van a consulta con un médico de vez en cuando a pesar de que se sienten bien y no han estado enfermas. ¿Cuándo fue la última vez que fuiste a consulta sintiéndote bien?	◯ Hace menos de un año<br>◯ Hace 1 - 2 años<br>◯ Hace 3 - 4 años<br>◯ Hace 5 o más años<br>◯ Nunca
7.3 ¿Cuándo fue la última vez que fuiste a consulta de odontología?	◯ Hace menos de un año<br>◯ Hace 1 - 2 años<br>◯ Hace 3 - 4 años<br>◯ Hace 5 o más años<br>◯ Nunca
7.4 ¿Qué tan seguido realizas el auto-examen testicular?	◯ Regularmente (2 - 4 veces al mes)<br>◯ Ocasionalmente(1 - 2 veces al mes)<br>◯ Raramente o nunca
7.5 ¿Qué tan seguido realizas el auto-examen de seno?	◯ Regularmente (2 - 4 veces al mes)<br>◯ Ocasionalmente(1 - 2 veces al mes)<br>◯ Raramente o nunca
7.6 ¿Cuándo fue tu última citología?	◯ Hace menos de un año<br>◯ Hace 1 - 2 años<br>◯ Hace 3 - 4 años<br>◯ Hace 5 o más años<br>◯ Nunca
7.7 ¿Cuándo fue la última vez que tuviste un examen de seno realizado por un médico o profesional de la salud?	◯ Hace menos de un año<br>◯ Hace 1 - 2 años<br>◯ Hace 3 - 4 años<br>◯ Hace 5 o más años<br>◯ Nunca
7.8 ¿Alguna vez te han realizado una mamografía?	◯ Si<br>◯ No
7.9 En caso de ser positivo, indica la edad aproximada cuando te la realizaron:	____________________________

85% Completado

VIII - Otras preocupaciones

Por favor indica qué tan preocupado(a) te sientes frente a las siguientes situaciones:

8.1 ¿Tu estado de salud en un futuro ?	◯ Muy preocupado(a)<br>◯ Un poco preocupado(a)<br>◯ Preocupado(a)<br>◯ No muy preocupado(a)<br>◯ Para nada preocupado(a)
8.2 ¿La posibilidad de tener hijos?	◯ Muy preocupado(a)<br>◯ Un poco preocupado(a)<br>◯ Preocupado(a)<br>◯ No muy preocupado(a)<br>◯ Para nada preocupado(a)
8.3 ¿Desarrollar un segundo cáncer?	◯ Muy preocupado(a)<br>◯ Un poco preocupado(a)<br>◯ Preocupado(a)<br>◯ No muy preocupado(a)<br>◯ Para nada preocupado(a)
8.4 ¿Tu capacidad de obtener un seguro de salud?	◯ Muy preocupado(a)<br>◯ Un poco preocupado(a)<br>◯ Preocupado(a)<br>◯ No muy preocupado(a)<br>◯ Para nada preocupado(a)
8.5 ¿Tu capacidad de obtener un seguro de vida?	◯ Muy preocupado(a)<br>◯ Un poco preocupado(a)<br>◯ Preocupado(a)<br>◯ No muy preocupado(a)<br>◯ Para nada preocupado(a)
8.6 ¿Cualquier otra situación que consideres preocupante?	◯ Si<br>◯ No
En caso de ser positivo, por favor especifica cuál:	__________________________
¿Te gustaría recibir chequeos médicos regulares (por ejemplo una vez al año) para revisar potenciales problemas a causa de tu cáncer en la infancia o adolescencia?	◯ Si<br>◯ No
Para continuar mejorando la atención para supervivientes de cáncer infantil o adolescencia, ¿qué tipo de información o ayuda consideras que debería estar disponible en el contexto del sistema de salud Colombiano?	________________________________

90% Completado

IX - CALIDAD DE VIDA

Las preguntas que siguen a continuación, se refieren a lo que piensas sobre tu salud. Tus respuestas permitirán saber cómo te encuentras y hasta qué punto eres capaz de hacer tus actividades habituales.

Contesta cada pregunta tal como se indica. Si no estás seguro/a de cómo responder a una pregunta, por favor contesta lo que te parezca más cierto.

9.1 En general, ¿dirías que tu salud es: (marca una sola opción)

- ◯ Excelente?
- ◯ Muy buena?
- ◯ Buena?
- ◯ Regular?
- ◯ Mala?

9.2 ¿Cómo calificarías tu estado de salud actual, comparado con el de hace un año? (Marca una sola opción)

- ◯ Mucho mejor ahora que hace un año
- ◯ Algo mejor ahora que hace un año
- ◯ Más o menos igual ahora que hace un año
- ◯ Algo peor ahora que hace un año
- ◯ Mucho peor ahora que hace un año

Las siguientes preguntas se refieren a actividades que tu puedes hacer durante un día normal. (Marca una opción en cada línea.)

	Si, me limita mucho	Si, me limita poco	No, no me limita para nada
9.3 Tu salud actual, ¿te limita para hacer actividades intensas, tales como correr, levantar objetos pesados, participar en deportes agotadores?	◯	◯	◯
9.4 Tu salud actual, ¿te limita para hacer actividades moderadas, tales como mover una mesa, empujar una aspiradora, trapear, lavar, jugar fútbol, montar bicicleta?	◯	◯	◯
9.5 Tu salud actual, ¿te limita para levantar o llevar las bolsas de compras?	◯	◯	◯
9.6 Tu salud actual, ¿te limita para subir varios pisos por las escaleras?	◯	◯	◯
9.7 Tu salud actual, ¿te limita para subir un piso por la escalera?	◯	◯	◯
9.8 Tu salud actual, ¿te limita para agacharte, arrodillarte o ponerte en cuclillas?	◯	◯	◯
9.9 Tu salud actual, ¿te limita para caminar más de un kilómetro (10 cuadras)?	◯	◯	◯

9.10 Tu salud actual, ¿te limita para caminar medio kilómetro (5 cuadras)?	◯	◯	◯
9.11 Tu salud actual, ¿te limita para caminar cien metros (1 cuadra)?	◯	◯	◯
9.12 Tu salud actual, ¿te limita para bañarte o vestirte?	◯	◯	◯

Las siguientes preguntas se refieren a problemas en tu trabajo a causa de tu salud física (Marca una opción en cada línea.)

	Si	No
9.13 Durante las últimas 4 semanas, ¿has disminuido el tiempo que dedicabas al trabajo o a tus actividades cotidianas, a causa de tu salud física?	◯	◯
9.14 Durante las últimas 4 semanas, ¿has hecho menos de lo que hubieras querido hacer, a causa de tu salud física?	◯	◯
9.15 Durante las últimas 4 semanas, ¿has tenido que dejar de hacer algunas tareas en tu trabajo o en tus actividades cotidianas, a causa de tu salud física?	◯	◯
9.16 Durante las últimas 4 semanas, ¿has tenido dificultades en realizar tu trabajo u otras actividades (por ejemplo, te ha costado más esfuerzo), a causa de tu salud física?	◯	◯

Las siguientes preguntas se refieren a problemas en tu trabajo a causa de algún problema emocional (como estar triste, deprimido/a o ansioso/a)
(Marca una opción en cada línea)

	Si	No
9.17 Durante las últimas 4 semanas, ¿has tenido que disminuir el tiempo que dedicabas al trabajo u otras actividades, a causa de algún problema emocional como estar triste, deprimido/a o ansioso/a?	◯	◯

9.18 Durante las últimas 4 semanas, ¿hiciste menos de lo que hubieras querido hacer, a causa de algún problema emocional (como estar triste, deprimido/a o ansioso/a)?	◯	◯
9.19 Durante las últimas 4 semanas, ¿has hecho el trabajo u otras actividades con menos cuidado de lo usual, a causa de algún problema emocional (como estar triste, deprimido/a o ansioso/a)?	◯	◯

9.20 Durante las últimas cuatro semanas, ¿en qué medida tu salud física o tus problemas emocionales han dificultado tus actividades sociales normales con tu familia, amigos, vecinos u otras personas?
(Marca una sola opción)

- ◯ Nada en absoluto
- ◯ Ligeramente
- ◯ Moderadamente
- ◯ Bastante
- ◯ Extremadamente

9.21 ¿Cuánto dolor físico has tenido durante las últimas cuatro semanas?
(Marca una sola opción)

- ◯ Ninguno
- ◯ Muy poco
- ◯ Poco
- ◯ Moderado
- ◯ Mucho
- ◯ Muchísimo

9.22 Durante las últimas cuatro semanas, ¿cuánto ha dificultado el dolor tu trabajo normal (incluyendo tanto el trabajo fuera del hogar como las tareas domésticas)?
(Marca una sola opción)

- ◯ Nada en absoluto
- ◯ Un poco
- ◯ Moderadamente
- ◯ Bastante
- ◯ Extremadamente

Las siguientes preguntas se refieren a cómo te has sentido y a cómo te han salido las cosas durante las últimas cuatro semanas. En cada pregunta, por favor elije la respuesta que más se aproxime a la manera como te has sentido.
(Marca una opción en cada línea.)

	Siempre	Casi siempre	Muchas veces	Algunas veces	Casi nunca	Nunca
9.23 ¿Cuánto tiempo durante las últimas cuatro semanas te has sentido lleno/a de vitalidad?	◯	◯	◯	◯	◯	◯
9.24 ¿Cuánto tiempo durante las últimas cuatro semanas has estado muy nervioso/a?	◯	◯	◯	◯	◯	◯

9.25 ¿Cuánto tiempo durante las últimas cuatro semanas te has sentido con el ánimo tan decaído/a que nada podría animarte?	○	○	○	○	○	○
9.26 ¿Cuánto tiempo durante las últimas cuatro semanas te has sentido tranquilo/a y sereno/a?	○	○	○	○	○	○
9.27 ¿Cuánto tiempo durante las últimas cuatro semanas has tenido mucha energía?	○	○	○	○	○	○
9.28 ¿Cuánto tiempo durante las últimas cuatro semanas te has sentido desanimado/a y triste?	○	○	○	○	○	○
9.29 ¿Cuánto tiempo durante las últimas cuatro semanas te has sentido agotado/a?	○	○	○	○	○	○
9.30 ¿Cuánto tiempo durante las últimas cuatro semanas te has sentido feliz?	○	○	○	○	○	○
9.31 ¿Cuánto tiempo durante las últimas cuatro semanas te has sentido cansado/a?	○	○	○	○	○	○

9.32 Durante las últimas cuatro semanas, ¿cuánto tiempo tu salud física o tus problemas emocionales han dificultado tus actividades sociales (como visitar amigos, parientes, etc.)?
(Marca una sola opción)

- ○ Siempre
- ○ Casi siempre
- ○ Algunas veces
- ○ Casi nunca
- ○ Nunca

Por favor, indica si te parece cierta o falsa cada una de las siguientes afirmaciones:
(Marca una opción en cada línea)

	Totalmente cierta	Bastante cierta	No sé	Bastante falsa	Totalmente falsa
9.33 Me parece que me enfermo más fácilmente que otras personas.	○	○	○	○	○
9.34 Estoy tan sano/a como cualquiera.	○	○	○	○	○
9.35 Creo que mi salud va a empeorar.	○	○	○	○	○
9.36 Mi salud es excelente.	○	○	○	○	○

95% Completado

Total Función Física

____

Total Desempeño físico

____

Total Dolor físico

____

Total Salud general

____

Total vitalidad

____

Total Función social

____

Total Desempeño emocional

____

Total salud mental

____

Total Cambio de salud

____

Total SF36

____

Comentarios

Para terminar, quisiéramos saber si tienes algún comentario adicional sobre el cuestionario y/o sobre tu experiencia como superviviente de cáncer infantil o adolescencia:	________________________________
También quisiéramos saber si te podemos contactar para futuros proyectos relacionados con este tema?	◯ Si<br>◯ No
En caso de ser positivo, te pedimos dejar los siguientes datos de contacto:<br><br>(Es importante aclarar que esta información se utilizará únicamente para fines de investigación y no se compartirán con terceros)	
Número de teléfono celular:	__________________________
Correo electrónico:	__________________________
Para terminar pulsa "SUBMIT"	
100% Completado	
